# Interplay between severities of COVID-19 and the gut microbiome: implications of bacterial co-infections?

**DOI:** 10.1186/s13099-021-00407-7

**Published:** 2021-02-25

**Authors:** Jyoti Chhibber-Goel, Sreehari Gopinathan, Amit Sharma

**Affiliations:** Molecular Medicine Group, International Center for Genetic Engineering and Biotechnology, New Delhi, India

**Keywords:** Angiotensin-converting enzyme 2, Gut-lung axis, Gut-microbiome, Opportunistic pathogens, Probiotics, SARS-CoV-2

## Abstract

COVID-19 is an acute respiratory distress syndrome and is often accompanied by gastrointestinal symptoms. The SARS-CoV-2 has been traced not only in nasopharyngeal and mid-nasal swabs but also in stool and rectal swabs of COVID-19 patients. The gut microbiota is important for an effective immune response as it ensures that unfavorable immune reactions in lungs and other vital organs are regulated. The human gut-lung microbiota interplay provides a framework for therapies in the treatment and management of several pulmonary diseases and infections. Here, we have collated data from COVID-19 studies, which suggest that bacterial co-infections as well as the gut-lung cross talk may be important players in COVID-19 disease prognosis. Our analyses suggests a role of gut microbiome in pathogen infections as well as in an array of excessive immune reactions during and post COVID-19 infection recovery period.

## Background

COVID-19 has been spreading all across the globe causing serious health concerns. COVID-19 patients have an altered gut microbiome. A proximal relationship between human gut and lungs has been established already, and microbial dysbiosis has been implicated in the susceptibility to bacterial infections. Here, we have collated data from COVID-19 studies that suggest that bacterial co-infections as well as the gut-lung cross talk may be important players in COVID-19 disease prognosis.

## Introduction

Severe Acute Respiratory Coronavirus 2 (SARS-CoV-2) is the causative driver of COVID-19 which has spread globally with serious public health consequences. The role of angiotensin-converting enzyme 2 (ACE2) in invasion of the host cells by SARS-CoV-2 via its spike protein is now established [1]. ACE2 receptor is part of the renin–angiotensin–aldosterone system (RAAS) that is pivotal in cardiovascular, renal and pulmonary diseases. It also has a crucial role in acute respiratory distress syndrome (ARDS) [2]. The ACE2 protein is cleaved by transmembrane proteases such as transmembrane protease serine 2 during SARS-CoV-2 entry thereby being down-regulated, resulting in severe pulmonary conditions and exaggerating the morbidities already present like those seen in COVID-19 patients [3]. This observation is supported by reports of reduced cardiac activity in ACE2 knockout mice and elevated levels of Angiotensin II (Ang II) in heart, kidneys and plasma [4]. Further, the reduction of ACE2 exacerbates kidney injury in diabetic mice, while treatment with ACE2 prevents heart injury, AngII induced hypertension and reverses diabetic nephropathy in murine model [5–7]. Evidently, individuals (n = 100) who were prescribed angiotensin converting enzyme inhibitor (ACEI) or angiotensin receptor blocker (ARB) present with a reduced risk of gastrointestinal (GI) complications and lower mortality [8]. SARS-CoV-2 infection reduces the expression of ACE2 in the GI tract and the number of circulating angiogenic cells (CACs) thus endangering the gut endothelium, leading to intestinal dysbiosis [9]. It is noteworthy that the number of ACE-2 receptors in the duodenum increase with age, suggesting a potential entry mechanism for the SARS-CoV-2 [10]. The above observations implicate a role of host gut in the infectivity and severity of COVID-19 infection [11].

## SARS-CoV-2 and host microbiome

SARS-CoV-2 has been found in nasopharyngeal, mid-nasal, stool and rectal swabs of COVID-19 patients [12–14]. ACE2 receptors are expressed in multiple organs including the gastrointestinal and respiratory tracts [15]. As stated previously, impaired ACE2 expression is associated with viral infection, immune imbalance as well as with intestinal bacterial dysbiosis, and is well established in SARS-CoV-2 [16]. Fecal metabolomic research has indicated possible amino acid-related pathways in COVID-19 patients that link gut microbiota to inflammation in COVID-19 patients [17]. For instance, ACE2 is found to stabilize the neutral amino acid (tryptophan) transporter B0AT1 (sodium-dependent neutral amino acid transporter) in the small intestine and regulate levels of gut anti-microbial peptides thereby maintaining gut stability [18]. Briefly, diet is the main source of tryptophan, whose uptake depends on B0AT1. ACE2 deficient mice lack B0AT1 in the small intestine and thereby have reduced plasma levels of the tryptophan [19]. Reduced levels of tryptophan result in failure to activate the secretion of antimicrobial peptides. This in turn results in inability to modulate the composition of intestinal bacterial population and subsequent susceptibility to inflammation of the large intestine [19]. Further to this, it has been established that COVID-19 patients with GI complications experience increased respiratory distress when compared to the patients without GI involvement [20, 21]. Gut bacterial dysbiosis has also been implicated in obesity, diabetes mellitus, cardiovascular diseases and several age related disorders [22]. People with these comorbidities as well as immune-compromised individuals are more vulnerable to COVID-19 with a manifold increase in their fatality rate [15, 23–25]. This is of particular interest based on evidence from previous studies that link gut microbiome and lung immunity [26]. Intestinal microbial dysbiosis modulates the immune responses of neutrophils, T-cell subsets, inflammatory cytokines, and Toll-like receptors thereby influencing pulmonary dysfunction [27–31]. Similarly, during an infection of the respiratory tract, the commensal microbes in the host lung stimulate local (lung) and distal (gut) immune responses [32]. These studies emphasize the importance of host microbiome during the course of an infection, either in the lung or gut. In the below sections, we focus on the gut bacterial population during- and post-COVID-19 infection period, while emphasizing its link with lung dysfunction and implications in infections from gut pathogens.

## Gut bacteria and cytokine storm

The impact of host microbiome on trajectories of both non-communicable and infectious diseases has been accepted [33, 34]. A role of gut bacteria in modulating the immune system of the host is also well established [35]. During COVID-19 infection, the pro-inflammatory cytokines IL-6, IL-10, IFN and TNF-α are raised in severe/critical patients [36]. The severity of COVID-19 is proposed to be a consequence of ‘cytokine storm’ [36]. It is noteworthy that several of the above cytokines are often correlated with the gut bacterial population and in initiating the cytokine storm (discussed in later sections). Further, via its association with Toll-like receptors, the gut bacteria are also an important factor in sustaining the cytokine storm [37].

Studies have shown that COVID-19 patients present with decreased levels of probiotic bacteria (e.g. *Lactobacillus* and *Bifidobacterium*) [38]. This can be taxing since high levels of *Lactobacillus* spp. correlate with increased anti-inflammatory IL-10 cytokine [39]. The cytokine marker IL-10 can possibly be used as a predictor for fast diagnosis of patients with higher risk of COVID-19 disease deterioration during the infection [40, 41]. Several gut commensals with known immunomodulatory potential such as *Faecalibacterium prausnitzii*, *Eubacterium rectale* and *bifidobacteria* were under represented in COVID-19 patients and remained low in samples collected up to 30 days after COVID-19 resolution [42]. Additionally, the abundance of butyrate-producing bacteria such as *Faecalibacterium prausnitzii*, *Clostridium butyricum*, *Clostridium leptum*, and *Eubacterium* was noted to decrease significantly [43]. COVID-19 cases were characterized by depletion of beneficial commensals like *Eubacterium ventriosum, Faecalibacterium prausnitzii, Lachnospiraceae taxa, Roseburia* and of *Bacteriodes* spp. like *B. dorei, B. massiliensis, B. ovatus* and *B. thetaiotaomicron* that correlate with the disease severity [44].

The gut flora dysbiosis during COVID-19 infection results in an overriding of the commensal bacterial population by pathogenic species. For instance, higher levels of *Klebsiella, Streptococcus,* and *Ruminococcus gnavus* in COVID-19 patients have been correlated with a surge in proinflammatory cytokines—(IFN-γ, TNF-α) leading to cytokine storm and activation of T helper (Th1) cell response [41]. These lead to increased COVID-19 severity in the patient cohort [41]. The opportunistic pathogens—*Streptococcus, Rothia, Veillonella, Erysipelatoclostridium* and *Actinomyces,* along with pro-inflammatory bacteria—*Erysipelotrichaceae bacterium 2_2_44A*,* Coprobacillus bacterium, Clostridium ramosum, Clostridium hathewayi* also increase during the course of COVID-19 disease in fecal samples (n = 15) [45]. The number of common opportunistic pathogens from the genus *Enterococcus,* phylum *Firmicutes* like *E. faecalis* and from the *Enterobacteriaceae* family which includes *Escherichia coli* and *Klebsiella pneumoniae* are increased in critically ill COVID-19 patients with poor prognosis [43]. This suggests that the gut bacterial composition and gut infection may play a role in regulating our immune responses during COVID-19.

## COVID-19 and gut pathogens

**Fig. 1:**
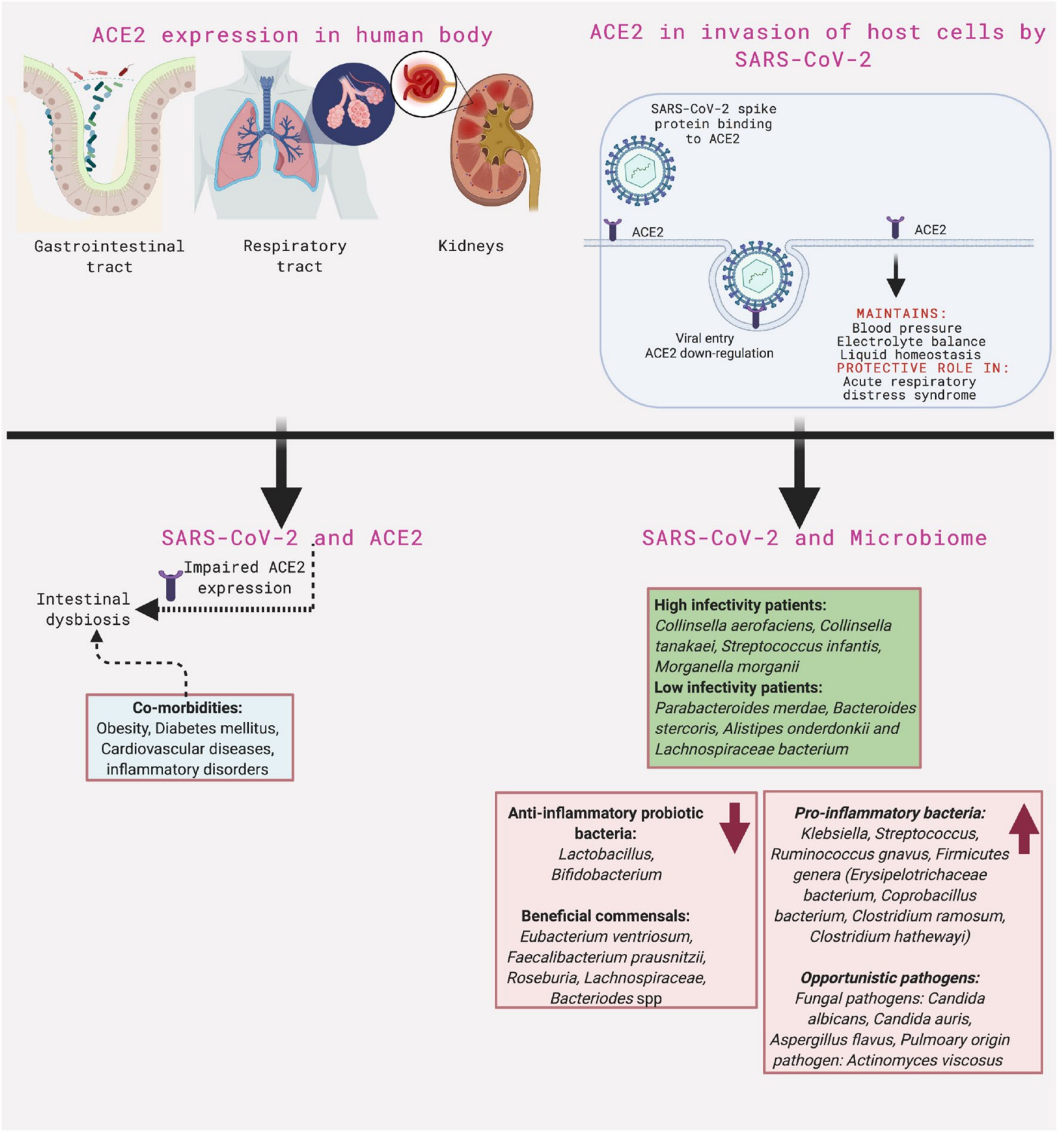
The interplay of human microbiome–SARS-CoV-2 in bacterial co-infections. The role of ACE2 in invasion of host cells by SARS-CoV-2 and the implications of host microbiome in bacterial co-infections during COVID-19 is shown

Data from more recent studies indicate that SARS-CoV-2 infection resolution can lead to persistent GI dysfunction that is similar to certain aspects of post-inflammatory gut-brain interaction disorder (GBID) and functional gastrointestinal disorder (FGID) [46]. This is attributed to the relationships between low-grade intestinal inflammation, increased permeability and dysbiosis together with environmental and psychological distress [46]. As stated in the previous section, the gut bacteria composition is modulated during the COVID-19 infection resulting in the susceptibility of host towards the pathogenic species and thus bacterial infections. In particular, a recent study showed that the fecal samples from COVID-19 patients tested positive for active coronavirus signatures up to 6 days after clearance of SARS-CoV-2 from the respiratory system [47]. Further, these fecal samples showed higher abundances of bacterial pathogenic species *Collinsella aerofaciens*, *Collinsella tanakaei*, *Streptococcus infantis* and *Morganella morganii* [47]. Amongst these species, *C. aerofaciens* results in loss of gut epithelial integrity via increasing the expression of pro-inflammatory cytokine IL-17 and of the chemokines CXCL1 and CXCL5 by intestinal epithelial cells [48]. While the role of *S. infantis* and its direct implication in gut diseases remains obscure, the levels of *S. infantis* are increased in individuals with acute cerebral infarction compared with healthy controls [49]. *M. morganii* is a rare opportunistic pathogen associated with multiple organ pathogenicity across all age groups [50]. On the other hand, the fecal samples that had low to none SARS-CoV-2 infectivity reported higher abundances of short-chain fatty acid (SCFA)-producing bacteria like *Parabacteroides merdae, Bacteroides stercoris, Alistipes onderdonkii* and *Lachnospiraceae bacterium* 1_1_57FAA [43]. SCFAs such as acetate, propionate and butyrate are important metabolites in maintaining intestinal homeostasis [51]. They are an important fuel for intestinal epithelial cells that are responsible for strengthening the gut barrier function [51]. Furthermore, compared with a group of adult controls, COVID-19 patients were observed to have more *Ruminococcus gnavus* (associated with Crohn’s disease), *Ruminococcus torques* (associated with C-reactive protein (CRP) +ve Crohn’s disease patients), and *Bacteroides dorei* (dominates gut microbiome prior to autoimmunity in patients at high risk type I diabetes) species, regardless of whether or not patients received medication [42]. COVID-19 patients also test positive for gastrointestinal pathogen *C. difficile* more often during the course of COVID-19 infection when compared to COVID-19 negative patients [52]. Aside from *C. difficile*, the other common enteric infections are attributed to *E. coli* species [52]. The increased presence of distinct opportunistic pathogens (like *Actinomyces viscosus*) of pulmonary origin in the gut insinuates a possible gut-lung interplay and demands further investigation [45]. These observations are worrisome since the effect of gut on immune system, gut-brain axis and several non-communicable diseases will influence recovery from COVID-19 and indeed may result in long term health complications (Fig. 1) [26]. Based on these, there is a need to explore the role and impact of the SARS-CoV-2 on the host microbiome in the gut during and post COVID-19.

## SARS-CoV-2 and probiotics

Probiotics are defined as live microorganisms that confer health benefits when they are administered in adequate amounts [53]. Clinical evidence has suggested that probiotics help in preventing GI diseases, diarrhea, sepsis as well as respiratory tract infections (RTIs) [54]. Randomized controlled trials have suggested that patients on mechanical ventilation develop significantly less ventilator-associated pneumonia compared to placebos when given probiotics (*Lactobacillus rhamnosus* GG, live *Bacillus subtilis*) [55]. The impact of diet, nutrients and probiotics in reducing the severity of COVID-19 infection has been suggested [56]. This is attributed to the probiotics resulting in the enrichment of “good bacteria” in the gut, by contributing to a change in the equilibrium between Th1/Th2 cells and thereby reducing the cytokine storm and hence the severity of COVID-19 [57]. It is however important to note that individuals with pre-existing clinical conditions (malignancies, leaky gut, diabetes mellitus, and post-organ transplant convalescence) likely fail to reap the benefits of probiotics. For instance probiotics have been implicated in systemic infections, pro-inflammatory immune responses, metabolic disorders as well as in horizontal gene transfer in bacteria resulting in opportunistic pathogenic infections [58]. Certain probiotic strains take advantage of the weak host immunity among the vulnerable groups and turn into opportunistic pathogens resulting in life-threatening pneumonia, endocarditis or sepsis. Thus the possible effects of diet and probiotics in modulating the severity of COVID-19 deserve more interrogation.

## Conclusion

In this analysis we have summarized the studies to date that provide evidence for bidirectional interactions between the gut and the lung via the host microbiome in COVID-19, with implications for post-COVID-19 GI complications (primary and secondary) as well as for gut infections. In summary, GI injury in COVID-19 patients can be divided into two groups—primary and secondary. In the former SARS-CoV-2 transmits to the GI tract by passing through the digestive system. The secondary GI injury is associated with pulmonary SARS-CoV-2 infection resulting from endothelial damage and thrombo-inflammation in the blood vessels, and/or by dysregulation of the immune system. Subsequently, GI injury may be exacerbated by gut microbial dysbiosis. In addition to the COVID-19-associated symptoms, an increased number of post-COVID-19 recovery symptoms are being reported worldwide. Since the human microbiome is adaptable and can be re-seeded with the help of dietary changes, we emphasize that studies on its impact on COVID-19 outcomes are needed currently.

## Data Availability

Not Applicable.

## Abbreviations

SARS-CoV-2: Severe acute respiratory corona virus 2
ACE 2: Angiotensin-converting enzyme 2
RAAS: Renin–angiotensin–aldosterone system
ARDS: Acute respiratory distress syndrome
Ang II: Angiotensin II
ACEI: Angiotensin converting enzyme inhibitor
ARB: Angiotensin receptor blocker
CRP: C- reactive protein
GBID: Gut–brain interaction disorder
FGID: Functional gastrointestinal disorder
GI: Gastrointestinal
CAC: Circulating angiogenic cells
RTI: Respiratory tract infections
SCFA: Short chain fatty acid
Th1: T helper 1
Th2: T helper 2
